# The Anti-Aging Potential of Neohesperidin and Its Synergistic Effects with Other Citrus Flavonoids in Extending Chronological Lifespan of *Saccharomyces Cerevisiae* BY4742

**DOI:** 10.3390/molecules24224093

**Published:** 2019-11-13

**Authors:** Chunxia Guo, Hua Zhang, Xin Guan, Zhiqin Zhou

**Affiliations:** 1 College of Horticulture and Landscape Architecture, Southwest University, Chongqing 400716, China; guochunxia@xdf.cn; 2 College of Biology and Food Engineering, Chongqing Three Gorges University, Chongqing 404120, China; 20140055@sanxiau.edu.cn; 3 Engineering Technology Research Center of Characteristic Biological Resources in Northeast of Chongqing, Three Gorges University, Chongqing 404120, China; 4 Key Laboratory of Horticulture for Southern Mountainous Regions, Ministry of Education, Chongqing 400715, China; 5 The Southwest Institute of Fruits Nutrition, Liang jiang New District, Chongqing 401121, China

**Keywords:** citrus flavonoids, neohesperidin, anti-aging activity, chronological lifespan, synergistic effect

## Abstract

The anti-aging activity of many plant flavonoids, as well as their mechanisms of action, have been explored in the current literature. However, the studies on the synergistic effects between the different flavonoid compounds were quite limited in previous reports. In this study, by using a high throughput assay, we tested the synergistic effects between different citrus flavonoids throughout the yeast’s chronological lifespan (CLS). We studied the effect of four flavonoid compounds including naringin, hesperedin, hesperitin, neohesperidin, as well as their different combinations on the CLS of the yeast strain BY4742. Their ROS scavenging ability, in vitro antioxidant activity and the influence on the extracellular pH were also tested. The results showed that neohesperidin extended the yeast’s CLS in a concentration-dependent manner. Especially, we found that neohesperidin showed great potential in extending CLS of budding yeast individually or synergistically with hesperetin. The neohesperidin exhibited the strongest function in decreasing the reactive oxygen species (ROS) accumulation in yeast. These findings clearly indicated that neohesperidin is potentially an anti-aging citrus flavonoid, and its synergistic effect with other flavonoids on yeast’s CLS will be an interesting subject for future research of the anti-aging function of citrus fruits.

## 1. Introduction

Aging, a complex and multifactorial biological process, can be defined as a gradual loss of physiological and psychological integrity, leading to gradual deterioration in almost all functions and the increased vulnerability to death [1]. A treatment that targets the multiple factors and/or pathways within the aging process is good candidate for study. At present, the trend of population aging is gradually increasing. Because of this trend in population, it is of great practical significance to find effective ways to slow aging or improve the healthy state of aging. The anti-aging activity of phytochemicals has been studied by the researchers from a wide variety of disciplines. Many different plant compounds have been suggested to have direct/potential anti-aging activity in the existing literature [2,3,4,5].

The budding yeast *Saccharomyces cerevisiae* has played a leading role as a model organism for studying evolutionarily conserved mechanisms, which are relevant to human aging and age-related diseases [6]. There are two types of lifespan in yeast, namely replicative lifespan (RLS) [7] and chronological lifespan (CLS) [8]. RLS is defined as the number of daughter cells a mother cell can produce before cell budding ceases [9], whereas CLS is the length of time budding yeast cells survive after undergoing a nutrient depletion-induced arrest of the cell cycle in stationary phase [10]. RLS and CLS can serve as models for proliferating and non-proliferating tissues in higher eukaryotes, respectively [6].

The longstanding and successful use of herbal drug combinations in traditional medicine inspired us to study the synergistic effect of phytochemicals that have healthy functions. Nowadays, synergy assessment has become a key area in medical research in order to enhance the efficiency of treatments and affect not only one single target, but several targets [11]. Previous investigations have shown that naringin, hesperidin, hesperetin and neohesperidin, widely distributed in citrus fruits, possess multiple biological activities relevant to anti-aging, and the detailed information of these phytochemicals are presented in literature [12,13,14,15,16,17,18,19,20,21]. However, the phytochemicals were less evaluated as combinations. At least, the ternary combinations of them were rarely reported. Meanwhile, the effect of neohesperidin on CLS of the budding yeast BY4742 was not revealed before.

In order to reveal the flavonoid combinations function on extending the CLS of the budding yeast BY4742, four flavonoid compounds, their binary and ternary combinations effect on CLS of yeast BY4742, and their ROS scavenging ability and in vitro antioxidant activity were also tested in the present work. Meanwhile, the extracellular pH values of yeast treated with the four compounds were detected since extracellular acidification of the culture medium might cause intracellular damage in the chronologically aging population [22].

## 2. Results

### 2.1. Neohesperidin Extended Yeast Lifespan in a Concentration-Dependent Manner

**Figure 1:**
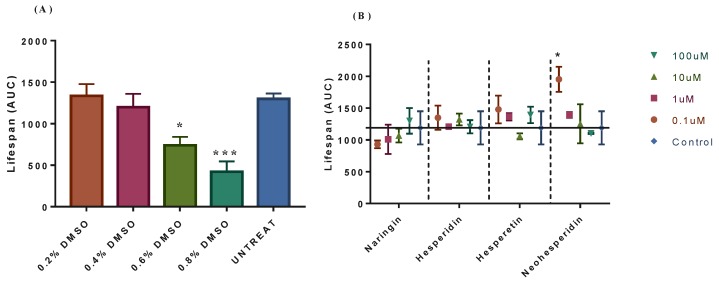
(**A**) Effect of different concentrations of dimethyl sulfoxide (DMSO) on lifespan of yeast BY4742. (**B**) Effect of different concentrations of the four flavonoid compounds on lifespan of yeast BY4742. AUC means area under the curve. The data was expressed as the mean values ± standard error of mean (SEM), *n* = 3. One-way ANOVA’s Sidak’s multiple comparisons test by GraphPad Prism 7.00 was used. (* *p* < 0.05, *** *p*< 0.001).

DMSO was universally used as a solvent for the water-insoluble drugs, and different concentrations of DMSO variably affected the growth of the yeast [23]. Therefore, we firstly screened the appropriate DMSO concentration. As shown in Figure 1 (A), there was no significant difference of yeast lifespan at 0.2% and 0.4% DMSO in the culture medium when compared with the control, while the lifespan at 0.6% and 0.8% DMSO were significantly decreased. To eliminate the influence of solvent in experiments, we set the final medium concentration of DMSO to 0.2% for carrying out the following experiments.

High-throughtput assays were used for rapid quantification of the CLS for the merit of the yeast chronological aging model [24,25]. Therefore, we employed this method to screen four citrus flavonoids (naringin, hesperidin, hesperetin and neohesperidin) for their anti-aging activity. We determined the longevity efficacy of the four phytochemicals in a range of doses, from 0 µM to 100 µM. From the result, it was found out that neohesperidin exhibited a potential to extend the CLS of BY4742 at 0.1 µM, while other three compounds could not increase the cell survival at the set concentrations (Figure 1B). For the convenience of combinatorial experiments, here the concentrations of A, B, C and D as 100, 0.1, 0.1 and 0.1 µM were chose, respectively. Namely, for the next combinational assays, A (100 µM naringin), B (0.1 µM hesperidin), C (0.1 µM hesperetin) and D (0.1 µM neohesperidin) were used.

**Figure 2:**
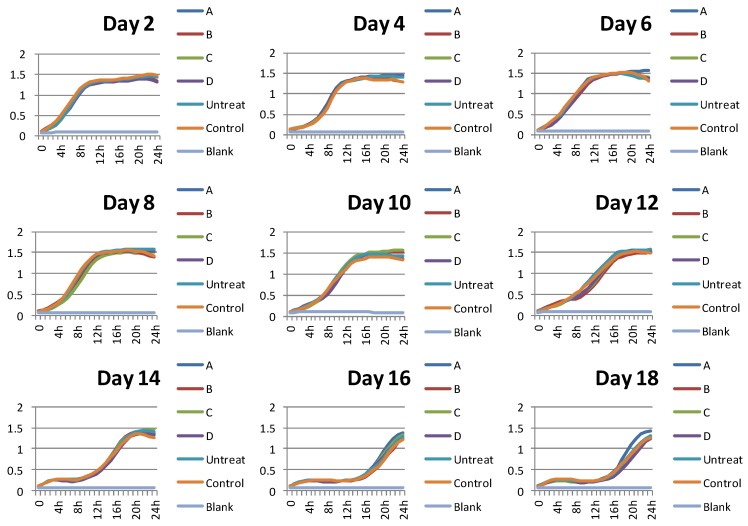
The growth curves of yeast BY4742 when treating with the four flavonoid compounds at different time at selected concentrations (100 µM A, 0.1 µM B, 0.1 µM C, and 0.1 µM D). The number of the day means the time of culturing in aging medium. And the growth curves were detected instantly after adding the compounds. The experiment was repeated three times. (A: naringin; B: hesperidin; C: hesperetin; D: neohesperidin).

**Figure 3:**
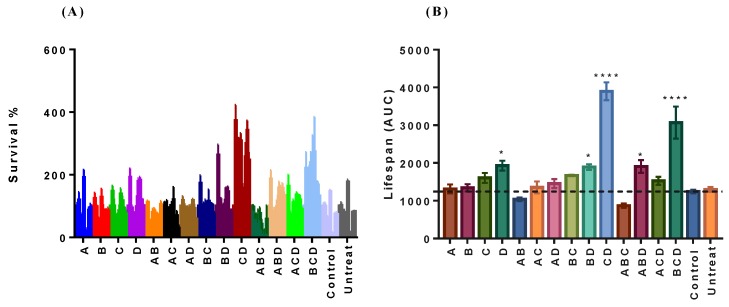
(**A**) The survival rates of yeast BY4742 under the treatment of different compounds and their combinations from day 2 to day 20, they were measured every two days. The survival rate was calculated as follows. Where tOD= 0.3, 2day is the time that OD value of day 2 age-point reaches 0.3 in the outgrowth curves. The initial age-point (day 2) is defined to be 100% viability and the relative survival percent of each successive age-point can be calculated as follows: $Vn=\frac{1}{2\frac{\Delta t_{n}}{\bar{Dt_{n}}}}\times 100n=days$, $\bar{Dt_{n}}$ represent the average doubling time. The survival integral (SI) for each well is defined as the area under the survival curve (AUC) and can be estimated by the formula: SI = $\sum_{2}^{n}\left(\frac{V_{n-1}+V_{n}}{2}\right)(day_{n}-day_{n-1})$, where *day_n_* is the age point, such as days 2, 4, 6, 8, 10, 12, 14, 16, 18 and 20. (**B**) The effect of the four flavonoid compounds, their binary and ternary combinations on chronological lifespan in yeast BY4742 at their beneficial concentrations. AUC means area under the curve. One-way ANOVA multiple comparisons. The data was expressed as the mean values ± standard error of mean (SEM), *n* = 4. * *p* < 0.05, **** *p* < 0.0001. A (100 µM naringin), B (0.1 µM hesperidin), C (0.1 µM hesperetin), D (0.1 µM neohesperidin).

The cumulative time of processing is thought to be an important factor for influencing cell growth. In accordance with this hypothesis, our data implied that the four flavonoid compounds did not influence the cell growth instantly after adding in 24 h at different processing times (Figure 2). Because the growth curves of yeast treating with A, B, C and D were almost the same as the control ones. This also showed that the four compounds did not inhibit the yeast cell growth. However, when the processing was started at day 2, the growth survival rates/CLS could change during long-lasting period (Figure 3).

### 2.2. Neohesperidin Positively Interacted with Hesperetin for Extending the CLS of Yeast BY4742

The combination therapy was demonstrated to be a new and highly effective therapeutic strategy to manage many diseases, such as diabetes [26], cancer [27], cardiovascular disorders [28], obesity and osteoporosis [29]. Here, we studied the effect of binary and ternary combinations of naringin, hesperidin, hesperetin and neohesperidin on the CLS of budding yeast BY4742. Figure 3A showed the survival rates of yeast under different flavonoid treatments. For each treatment, 10 aging points were detected. It showed that the survival rates of some treatment were higher when compared to the counterparts of control. From Figure 3B, the result clearly showed us that the treatment of D, BD and ABD showed the significant differences (*p* < 0.05), just weaker than CD and BCD (**** *p*< 0.0001). We can see that D (neohesperidin) showed important function in the individual or synergistical treatments. Therefore, we could conclude that neohesperidin had great potential in increasing CLS of budding yeast BY4742 individually or synergistically with hesperetin.

### 2.3. Neohesperidin Significantly Reduced Intracellular Reactive Oxygen Species (ROS) Content

**Figure 4:**
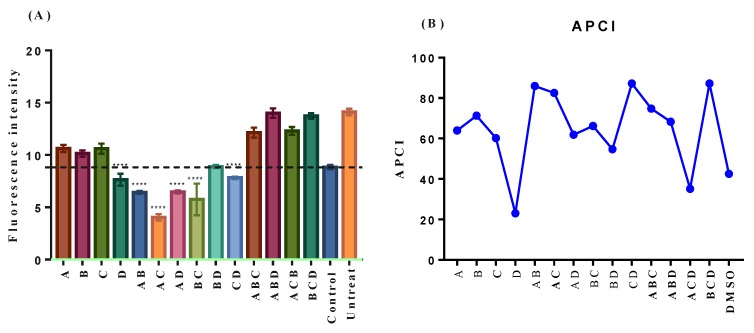
(**A**) Effect of A (100 µM naringin), B (0.1 µM hesperidin), C (0.1 µM hesperetin), D (0.1 µM neohesperidin), their binary and ternary combinations on intracellular ROS levels of yeast (BY4742) grown in standard SD medium after treating for 2 days (n = 12). The ROS probe H2DCFDA was used. Dichlorodihydrofluorescein (DCF). One-way ANOVA multiple comparisons. The data was expressed as the mean values ± standard error of mean (SEM), n = 12. **** *p* < 0.0001; (**B**) The antioxidant capacity (ÿ M trolox equivalents/µM phytochemical) of A (naringin), B (hesperidin), C (hesperetin), D (neohesperidin) and their binary and ternary combinations evaluated by 1,1-diphenyl-2-picrylhydrazyl (DPPH), FRAP and ABTS assays. APCI (antioxidant potency composite index) = Σ(the sample data of each method/the highest sample data of every method)/the number of methods •100. The higher the APCI is the lower the rank number is.

According to Harman, cellular component damage caused by ROS generated in mitochondria is the main force accelerating the aging process of the organism [30,31]. As shown in Figure 4A, the four flavonoids all exhibited a remarkable ROS scavenging capacity. Among four single treatments, neohesperidin had the most prominent effect. Other treatments combined, all the ternary combination, and BD as well, didn’t decrease intracellular ROS. As for the binary combinations, such as AB, AC, AD, and BC, they showed higher ROS scavenging activities than their corresponding single substances. The ROS scavenging capability of the binary combination BD was between B and D, while the function of CD was almost the same as D.

### 2.4. In Vitro Antioxidant Activity of Neohesperidin was Relatively Weak

Many methods are available for measuring the in vitro antioxidant capacity and most researchers apply one or more assays since each method measures different antioxidant characteristics of the compound [32]. In this study, we used three methods to determine antioxidant capacity include DPPH, ABTS, and FRAP assays. The antioxidant potency composite index (APCI) was defined to describe and evaluate the overall in vitro antioxidant capacity of the tested compounds and their combinations. The linear regression equations of the three assays are listed in Table 1. And all the related data were presented in Table 2. Meanwhile, the APCI was plotted in Figure 4B. From these results, we could see that neohesperidin had relatively weak in vitro antioxidant capacity; this implied the CLS extension function of neohesperidin was not depending on its in vitro antioxidant activity. However, with comparatively high in vitro antioxidant activity, CD and BCD extended the CLS of yeast BY4742. Overall, we cannot forecast a compound’s CLS extending capacity just based on its antioxidant activity.

### 2.5. Neohesperidin Could Not Slow Down the Variation of Extracellular Acidification of Yeast BY4742

**Figure 5:**
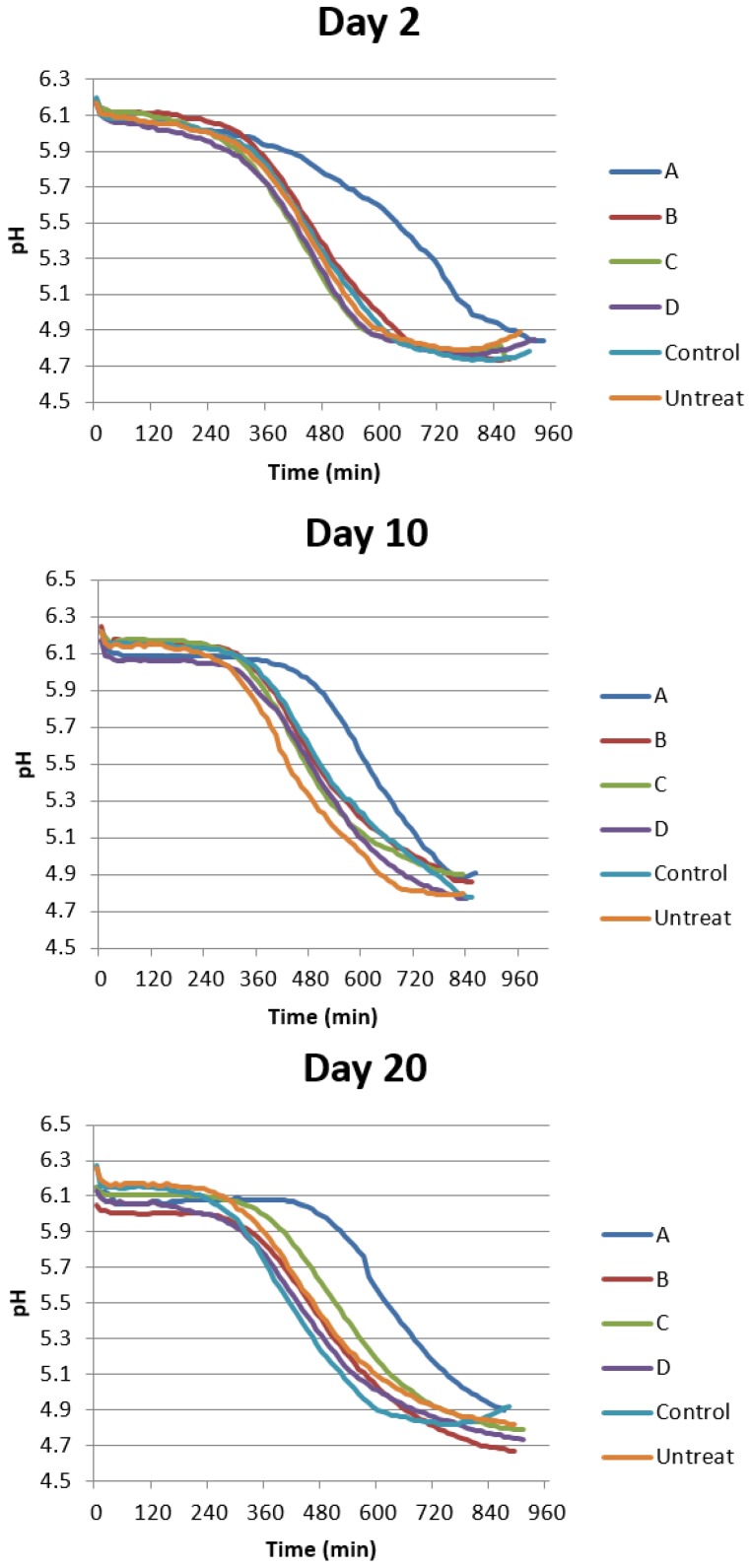
Variation of the pH of the culture mediums after the budding yeast BY4742 treated by the four flavoniod compounds at 10 µM. The experiment was performed at least in triplicate. A: naringin, B: hesperidin, C: hesperetin, D: neohesperidin.

Important parameters include the composition of the growth medium as well as the pH value. The composition of the growth medium and pH value has been shown to have major impact on the CLS of S. cerevisiae [33]. The effects of pH on CLS of budding yeast were investigated by previous studies, and the results point to a mechanism of acetic acid toxicity related to the induction of growth signaling pathways and oxidative stress in yeast [34]. In order to know the effect of the four flavonoid compounds on the variation of extracellular acidification of yeast cultures, we detected the pH values every five minutes using a pH meter. In Figure 5, 10 µM naringin obviously slowed down the variation of extracellular acidification of budding yeast BY4742 at different aging states while the other three flavonoid compounds did not influence it significantly at the same concentration when compared to control groups.

## 3. Discussion

Former studies had reported that neohesperidin exhibited various anti-aging associated functions, such as the neuroprotective effect [15], ROS-scavenging and anti-inflammatory activities [35], attenuation of the decrease of mitochondrial membrane potential and the increase of caspase-3 activity evoked by H_2_O_2_ [16], and cellular apoptosis-inducing activity [21]. All these functions laid a good foundation for the result that neohesperidin increased the CLS of budding yeast BY4742 here. It is surprising that neohesperidin extended the CLS significantly only at the lowest concentration tested. In the report of Craker, et al. it also showed a lower concentration of auxin (10-6 IAA) promotes proton-extrusion. Proton-extrusion under a high concentration of auxin (10-4 IAA) is inhibited by auxin-induced ethylene [36]. The ROS-scavenging activity of neohesperidin was verified in our research. The in vitro antioxidant capacity of neohesperidin was relatively weak, which explained why the CLS extension function of neohesperidin did not depend on its in vitro antioxidant activity. However, the combinations CD and BCD had high in vitro antioxidant activity and increased the CLS of yeast (Figure 3B). The weak correlation of ROS and antioxidant activity may be caused by the method we used to analyze the antioxidant activity. The antioxidant activity method tried to reacted with a double bond at C 2–C 3 and/or a hydroxyl group at C 3 on the C ring of flavonoid. In Areias et al. the results strongly suggested that the higher antioxidant activity of the flavonoids is not correlated with the presence of a double bond at C 2–C 3 and/or a hydroxyl group at C 3 on the C ring, but rather may depend on the capacity to inhibit the production of reactive oxygen species to interact hydrophobically with membranes [37]. At the same times, a large number of studies have shown that some antioxidants do have the function of extending lifespan, their specific mechanisms of action are complex. Only some antioxidants have been shown to exhibit anti-aging effects related to the direct free radical and ROS clearance. But the life-extending effects of other antioxidants on model organisms were not limited to direct antioxidant function, but also include the regulation of stress-related genes expression and the induction of toxic stimulatory effects [38]. Therefore, it is impossible to predict the ability of a substance to extend the CLS of yeast based on its antioxidant activity. Though we did not test the result in other strains, it offered information for other researchers and scientists to validate the result in other strains and model organisms.

Many cellular processes and extrinsic factors negatively influence the yeast chronological lifespan, including medium acidification andoxidative stress [39]. One of the early changes that occurs in yeast cells grown in media containing 2% glucose (dextrose) is the production of acetic acid and acidification of the medium, which has been shown to influence chronological aging [38]. Buffering the medium to pH 6–7 prevents acidification and increases chronological life span [10,39,40,41]. Additionally, acetic acid can be utilized by *Saccharomyces cerevisiae* for growth and metabolism in spite of its potential toxicity [42]. 10 µM neohesperidin, hesperidin and hesperetin maintained the variation trend of extracellular pH values when compared with control (Figure 5). However, 10 µM naringin clearly slowed down the variation of extracellular acidification (Figure 5). At this concentration, the CLS of yeast BY4742 was treated with neohesperidin, hesperidin and hesperetin were almost the same as the control group.

Increased ROS scavenging has marked effects on CLS in yeast, but the reason remains an unresolved issue [33]. Aging and related diseases are the consequence of free radical-mediated damage to cellular macromolecules and their inability to counterbalance endogenous antioxidant defenses mechanisms [43]. However, data has indicated that ROS also can play a positive role in inducing stress response genes (hormesis) [43,44,45,46].

Moreover, recent findings suggest that also the type of ROS and the time they occur are important for lifespan extension in *S. cerevisiae* [47,48,49], which illustrates the complex role of ROS in yeast aging. Graziano et al. [47] reported that neohesperidin decreased ROS generation in human keratinocytes. Nohara, et al. recently revealed that nobiletin (one of flavonoids in citrus) fortifies mitochondrial respiration in skeletal muscle to promote healthy aging against metabolic challenge. ROS production was significantly suppressed by nobiletin treatment in a dose-dependent manner [50]. From Figure 3B and Figure 4A, it showed that D and CD increased the CLS of yeast BY4742 and decreased the intracellular ROS content. But, for ABD and BCD, they prolonged the CLS while increased the intracellular ROS. This result was consistent with Wu’s [51]. So, there was no certain positive or negative relationship between ROS scavenging activities of the compounds and their effects on lifespan, and this was also in line with the intricate role of ROS in yeast aging.

In Figure 3A, the survival rates of yeast BY4742 under the treatment of different compounds and their combinations from day 2 to day 20 were not gradually decreasing. They present double peaks. This can be also found in the result of Wu et al. (2014). For the first few days, the survival rates were relatively high. This can be explained by the enough nutrient and low survival pressure during this time. As time went on, valley points appeared for a very short time as the nutrition became less. Then, peaks appeared again. This phenomenon might attribute the success to the metabolism of another nutrient that alleviated the survival pressure.

Qi et al. [36] reported that the antioxidant activity of antioxidants mixture/compounds combination was more effective than a single compound. In our experiment, the combinations AB, AC, CD, ABC, and BCD had a stronger antioxidant capacity than any single substance corresponding to them. Based on our observations, it could be concluded that the flavonoids present in a mixture could interact, and their interactions could affect the total antioxidant capacity of a solution (Figure 4B). Although we demonstrated that the four flavonoid interactions trigger synergistic or antagonistic effects for the antioxidant power, there are other flavonoid combinations that require a more detailed study in order to better understand the mechanisms involved in these interactions. Lutchman et al. [52] had reported plant extracts that increased the yeast ‘s CLS. And the autophagy promoted by decreased TORC1 signaling is critically important for a long CLS [10]. By referring to these studies, we can explore the way by which the compounds execute their effect in future studies.

## 4. Materials and Methods

### 4.1. Materials

The wild-type strain *S. cerevisiae* BY4742 (ATCC^®^, 201389^TM^) (*MATα his3*Δ1 *leu2*Δ0 *lys2*Δ0 *ura3*Δ0) was obtained from American Type Culture Collection (Manassas, VA, USA). The culture of yeast reference strain was aliquoted into 10 μL and stored at −80 °C. All L-amino acids, yeast nitrogen base w/o amino acids (YNB), ammonium sulfate, peptone, agar and yeast extract, H_2_DCFDA, 2,4,6-tripyridyl-s-triazine (TPTZ), 2,2′-azino-bis (3-ethylbenzothiazoline-6-sulfonic acid) (ABTS), 1,1-diphenyl-2-picrylhydrazyl (DPPH), dimethyl sulfoxide (DMSO), neohesperidin, naringin, hesperidin and hesperetin were bought from Sigma-Aldrich (Shanghai, China). YPD Broth, YPD and other chemicals were from Solebo biotech Co., Ltd. (Beijing, China). The 96-well polystyrene microplates with flat bottom were purchased from Corning incorporated (Kennebunk, ME, USA).

### 4.2. Lifespan and Yeast Cell Growth Assay

The determination of chronological lifespan (CLS) of yeast was carried out according to the method of Wu et al. [25] with a moderate modification as follows. In brief, the yeast cells were prepared by transferring a streaked strain from frozen stocks onto YPD agar (0.5% yeast extract/1% peptone/2% dextrose/1.4% agar) plates. After incubating the cells at 30 °C for 2 days, a single colony was picked and inoculated into a 1.0-mL YPD liquid medium (1% yeast extract/2% peptone/2% dextrose) in a 10-mL sterilized centrifuge tube (round bottom) and cultured at 30 °C for 2 days in a flat incubator at 200 rpm. The 2-day YPD culture was diluted with autoclaved 18 mΩ Milli-Q grade water (1:10) and stored in a refrigerator at 4 °C for 2 days. After 2-day incubation at 4 °C, 5 µL (≈ 1 × 10^4^ cells) of the diluted culture was transferred to 993 microliter of synthetic-defined (SD) media (Supplementary Table S1, [51]) and maintained at 30 °C, 200 rpm for the entire experiment. Compounds in DMSO with several concentrations (2.0 μL) were added at the initial inoculation (0 h). Each experiment was performed at least in triplicate. Cell cultures were incubated at 30 °C without replacing the aging medium throughout the experiment. After 2 days of culture in an aging media, the cells reached stationary phase and the first age-point was then taken. Subsequent age-points were taken every 2–4 days. For each age point, 5.0 μL of the mixed culture was pipetted into each well of 96-well flat-bottom microplate. Ninety-five microliter YPD medium was then added to each well. The cell population was monitored with a microplate reader (Varioskan Flash; Thermo Scientific, Waltham, MA, USA) by recording OD660 every 10 min during 24 h.

The survival rate was calculated as follows [25]. Where tOD= 0.3, 2day is the time that OD value of day 2 age-point reaches 0.3 in the outgrowth curves. The initial age-point (day 2) is defined to be 100% viability and the relative survival percent of each successive age-point can be calculated as follows:

$$Vn=\frac{1}{2\frac{\Delta t_{n}}{\bar{Dt_{n}}}}\times 100n=days,\;\bar{Dt_{n}}\;\mathrm{represent}\;\mathrm{the}\;\mathrm{average}\;\mathrm{doubling}\;\mathrm{time}.$$

The survival integral (SI) for each well is defined as the area under the survival curve (AUC) and can be estimated by the formula:

$$\mathrm{SI}\;=\;\sum_{2}^{n}\left(\frac{V_{n-1}+V_{n}}{2}\right)(day_{n}-day_{n-1})$$

where *day_n_* is the age point, such as days 2, 4, 6, 8, 10, 12, 14, 16, 18 and 20.

### 4.3. Intracellular ROS Scavenging Ability Assays

To quantify the intracellular reactive oxygen species (ROS) level of yeast cells grown in standard SD medium with/without the four compounds, the method described in Wu et al. [51] had been referenced. Namely, 2 μL ROS probe H_2_DCFDA from a fresh 5-mM stock solution in DMSO was added into 1.0 mL yeast aging culture (at day 2) at 30 °C for 1 h. The culture was then washed twice in sterile distilled water and suspended in 1.0 mL of 50 mM Tris/Cl buffer (pH 7.5). Twenty microliter of chloroform and 10 μL of 0.1% (*w*/*v*) sodium dodecyl sulfate (SDS) were added, and the cells were incubated at 200 rpm for 30 min to allow the dye to diffuse. The culture was centrifuged at 5000 rpm for 5 min, and the fluorescence of the supernatant was measured using a microplate reader with excitation at 480 nm and emission at 520 nm.

### 4.4. Antioxidant Activity Assays

In this study, DPPH, FRAP and ABTS assays were used for in vitro antioxidant capacity evaluation. The DPPH assay was performed according to the method described by Barreca et al. [53]. An aliquot of each sample (0.5 mL) was mixed with 75 μM (3.5 mL) of DPPH in methanol to a final volume of 4.0 mL. After reacting for 30 min without light, the absorption of the mixture was detected at a wavelength of 517 nm. The inhibition percentage of radical scavenging activity was the DPPH value. The FRAP assay was carried out according to Hungder et al. [54] with some modifications. A 0.2 mL aliquot of the sample was mixed with 3.8 mL of FRAP reagent (0.3 mol/L acetate buffer (pH 3.6), 10 mmol/L TPTZ solution, and 20 mmol/L ferric chloride (FeCl_3_) were mixed (10:1:1, volume ratio)). After 30 min, the absorbance was detected at wavelength of 593 nm. And the ABTS assay was followed the method of Mnb et al. [55] with few modifications. The ABTS radical cation (ABTS •+) was generated by reaction of 176 μL of potassium persulfate solution (140 mM) and 10 mL aqueous ABTS solution (7 mM) under the condition of no light for 12–16 h. Then it was diluted with methanol to an absorption value of 0.7 ± 0.02 units at 734 nm. 0.1 mL sample was added to 4.9 mL ABTS reagent. The absorbance was measured at a wavelength of 734 nm after 10 min reaction. All absorbance values were determined by using the UV–VIS spectrophotometer (PerkinElmer Lambda 25 UV/VIS, Waltham, MA, USA). Antioxidant values were calculated by standard curve method and expressed as trolox equivalents (TE mg/g DW).

### 4.5. Extracellular pH Detection

The culture process of yeast in the early stage was almost the same as the method described in the part of “Lifespan and yeast cell growth assay”. The specific operation was as follows. The yeast cells were prepared by transferring the yeast strain from frozen stocks onto YPD agar plates. After incubating the cells at 30 °C for 2 days, a single colony was picked and inoculated into a 1.0-mL YPD liquid medium in a 10-mL sterilized centrifuge tube and cultured at 30 °C for 2 days in a flat incubator at 200 rpm. The 2-day YPD culture was diluted with autoclaved 18 mΩ Milli-Q grade water (1:10) and stored in a refrigerator at 4 °C for 2 days. After 2-day incubation at 4 °C, 10 µL (≈ 2 × 10^4^ cells) of the diluted culture was transferred to 1986 µL of SD media and maintained at 30 °C, 200 rpm for 2/10/20 days. Various compounds in DMSO (4.0 μL) were add to the medium to a final concentration of 10 µM at initial inoculation (0 h). Then 1mL of the 2/10/20-day SD culture was added to 19 mL fresh YPD liquid medium. The pH was tested every five minutes using a pH meter while the yeast was cultured at 30 °C in a shaker at 200 rpm for the whole detection. Each experiment was performed at least in triplicate.

### 4.6. Data Analysis

The raw data from the microplate reader were exported to Microsoft Excel 2007 (Redmond, WA, USA). From the growth curves, the viability of the yeast can be obtained according to previous report [25]. Survival integral [56] of each aging culture was defined as the area under the survival curves [25,57]. The data were analyzed by one-way analysis of variance (one-way ANOVA), and were expressed as the mean values ± standard error of mean (SEM). The significance of difference (* *p* < 0.05; ** *p* < 0.01; *** *p* < 0.001; **** *p* < 0.0001) was determined using Sidak’s multiple comparisons test. GraphPad Prism 7 (GraphPad Software, Inc., La Jolla, CA, USA) was used for the analysis.

## 5. Conclusions

In conclusion, neohesperidin, with relatively high capability of removing intracellular ROS, showed great potential in extending the CLS of budding yeast BY4742 individually or synergistically with hesperetin. This might lead to new choices for the treatment of aging problems and since there was a limited relationship between the CLS-extension of yeast and the tested indexes (e.g., ROS scavenging ability, in vitro antioxidant activity and the extracellular pH). Further studies to discover the molecular mechanisms of this phenomenon will be extremely beneficial to prevent aging. Because of this, the importance of choosing the best combination of flavonoids, should be borne in mind when designing new dietary supplements or functional foods.

## Figures and Tables

**Table 1:** The linear regression equations of the three assays.

Method	Equation	R^2^	The Linear Range
DPPH	y = −0.0029x + 0.6716	0.9997	0–200 µM
ABTS	y = −0.0006x + 0.644	0.9962	0–1000 µM
FRAP	y = 0.0023x − 0.003	0.9992	0–560 µM

**Table 2:** The antioxidant capacities (µM trolox equivalents/µM phytochemicals) of the bioactive compounds analyzed in this study.

Phytochemicals	DPPH	ABTS	FRAP	APCI	Rank
**A**	11.33 ± 0.15 ^j^	550.18 ± 1.21 ^a^	350.74 ± 1.21 ^b^	63.96	9
**B**	33.31 ± 1.16 ^h^	533.57 ± 0.2 ^a^	302.64 ± 0.82 ^e^	71.32	6
**C**	41.39 ± 0.16 ^f^	447.33 ± 0.59 ^b^	160.01 ± 0.29 ^h^	60.17	10
**D**	28.74 ± 0.17 ^i^	119.33 ± 0.36 ^c^	7.54 ± 0.21 ^i^	23.02	15
**AB**	35.77 ± 0.06 ^g^	571.97 ± 7.13 ^a^	453.54 ± 0.86 ^a^	85.97	3
**AC**	45.8 ± 0.24 ^e^	548.94 ± 1.99 ^a^	350.96 ± 0.46 ^b^	82.51	4
**AD**	34.13 ± 0.10 ^h^	500.91 ± 1.03 ^b^	194.44 ± 0.62 ^g^	61.90	10
**BC**	53.74 ± 0.23 ^c^	432.93 ± 0.6 ^b^	162.59 ± 0.71 ^h^	66.19	8
**BD**	52.61 ± 0.05 ^c^	444.8 ± 27.13 ^b^	5.52 ± 0.22 ^i^	54.73	12
**CD**	58.52 ± 0.06 ^b^	546.17 ± 1.01 ^a^	323.97 ± 0.04 ^c^	87.23	1
**ABC**	58.61 ± 0.10 ^b^	477.95 ± 3.37 ^b^	208.58 ± 1.19 ^f^	74.82	5
**ABD**	47.58 ± 0.12 ^d^	489.66 ± 0.64 ^b^	191.63 ± 1.50 ^g^	68.30	7
**ACD**	61.75 ± 0.30 ^a^	25.05 ± 0.74 ^d^	4.03 ± 0.08 ^i^	35.09	14
**BCD**	61.23 ± 0.11 ^a^	538.31 ± 0.24 ^a^	310.05 ± 0.52 ^d^	87.21	2
**DMSO**	3.42 ± 0.18 ^k^	455.56 ± 0.54 ^b^	192.37 ± 0.26 ^g^	42.53	13

A: naringin, B: hesperidin, C: hesperetin, D: neohesperidin, the concentration of A, B, C and D is 1µM. APCI = Σ (each sample value/the biggest sample value in that method)/the number of methods. Data were expressed as mean ± SEM (*n* = 9) and compared using one-way ANOVA’s Sidak’s multiple comparisons test at *p* < 0.05 by GraphPad Prism 7.00. Different letters (a, b, c, d, e, f, g, h, i, j, k) after data indicate values in the same column significant differences.

## Supplementary Materials

The following are available online at https://www.mdpi.com/1420-3049/24/22/4093/s1, Table S1: Composition of synthetic-defined (SD) medium used for yeast chronological lifespan analysis.
