# Supplementary material for: The Anti-Aging Potential of Neohesperidin and Its Synergistic Effects with Other Citrus Flavonoids in Extending Chronological Lifespan of Saccharomyces Cerevisiae BY4742
Source: Molecules. 2019 Nov 13;24(22):4093. doi: 10.3390/molecules24224093 (PMC6891605; doi:10.3390/molecules24224093)
Supplement: Supplementary file 1 [file molecules-24-04093-s001.pdf]

Supplementary Table 1 Composition of synthetic-defined (SD) medium used for yeast chronological lifespan analysis. The table was quoted from Wu et al. [48].

**Component**	**Concentration**
Glucose.	20 g/L..
Yeast nitrogen base(−AA/−AS).	1.7 g/L..
Ammonium sulfate.	5 g/L..
Amino acids (×1)..	
**Essential..**	
Uracil.	100 mg/L..
L-Histidine.	10.<br>0 mg/L..
L-Leucine.	300 mg/L..
L-Lysine-HC1.	150 mg/L..
**Non-essential..**	
Adenine.	80 mg/L..
L-Arginine.	40 mg/L..
L-Aspartic acid.	100 mg/L..
L-Glutamic acid.	100 mg/L..
L-Methionine.	80 mg/L..
L-Phenylalanine.	50 mg/L..
L-Serine.	400 mg/L..
L-Threonine.	200 mg/L..
L-Tryptophan.	200 mg/L..
L-Tyrosine.	40 mg/L..
L-Valine.	150 mg/L..
L-Isoleucine.	60 mg/L..
